# Calculating Photoabsorption Cross-Sections for Atmospheric Volatile Organic Compounds

**DOI:** 10.1021/acsearthspacechem.1c00355

**Published:** 2021-12-17

**Authors:** Antonio Prlj, Emanuele Marsili, Lewis Hutton, Daniel Hollas, Darya Shchepanovska, David R. Glowacki, Petr Slavíček, Basile F. E. Curchod

**Affiliations:** † Department of Chemistry, Durham University, Durham DH1 3LE, U.K.; ‡ Department of Physical Chemistry, University of Chemistry and Technology, Prague, Technická 5, Prague 16628, Czech Republic; § Centre for Computational Chemistry, School of Chemistry, University of Bristol, Bristol BS8 1TH, U.K.; ∥ ArtSci International Foundation, 5th Floor Mariner House, Bristol BS1 4QD, U.K.; ⊥ CiTIUS Intelligent Technologies Research Centre, Rúa de Jenaro de La Fuente, s/n, Santiago de Compostela 15705, A Coruña, Spain

**Keywords:** computational photochemistry, photoabsorption cross-section, volatile organic compounds, atmospheric chemistry, quantum chemistry

## Abstract

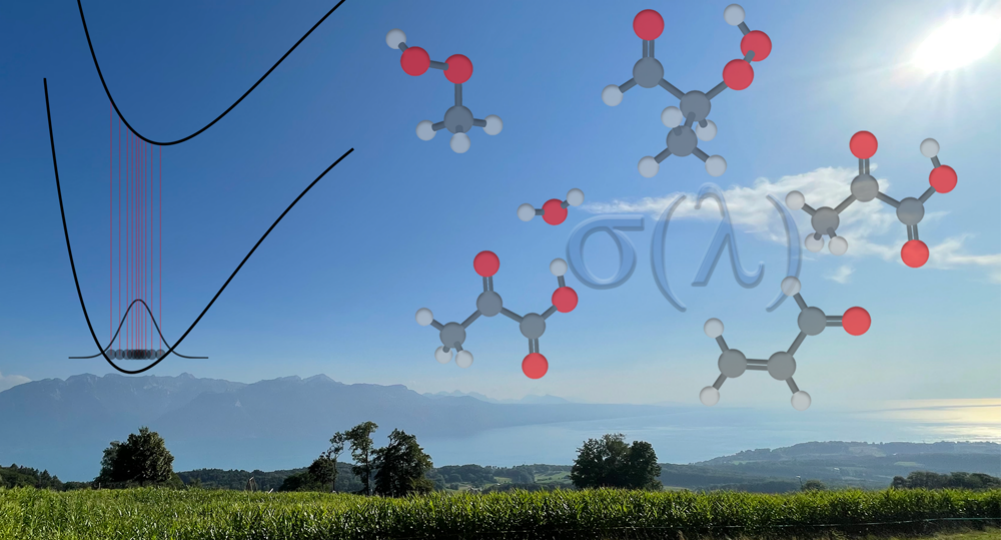

Characterizing the photochemical reactivity of transient volatile organic compounds (VOCs) in our atmosphere begins with a proper understanding of their abilities to absorb sunlight. Unfortunately, the photoabsorption cross-sections for a large number of transient VOCs remain unavailable experimentally due to their short lifetime or high reactivity. While structure–activity relationships (SARs) have been successfully employed to estimate the unknown photoabsorption cross-sections of VOCs, computational photochemistry offers another promising strategy to predict not only the vertical electronic transitions of a given molecule but also the width and shape of the bands forming its absorption spectrum. In this work, we focus on the use of the nuclear ensemble approach (NEA) to determine the photoabsorption cross-section of four exemplary VOCs, namely, acrolein, methylhydroperoxide, 2-hydroperoxy-propanal, and (microsolvated) pyruvic acid. More specifically, we analyze the influence that different strategies for sampling the ground-state nuclear density—Wigner sampling and ab initio molecular dynamics with a quantum thermostat—can have on the simulated absorption spectra. We highlight the potential shortcomings of using uncoupled harmonic modes within Wigner sampling of nuclear density to describe flexible or microsolvated VOCs and some limitations of SARs for multichromophoric VOCs. Our results suggest that the NEA could constitute a powerful tool for the atmospheric community to predict the photoabsorption cross-section for transient VOCs.

## 1 Introduction

UV/vis linear absorption spectroscopy is one of the most fundamental techniques to investigate molecular optical properties. A photoabsorption spectrum or cross-section not only is the fingerprint of a given molecule but also underlies many of the exciting photophysical and photochemical processes a given molecule can undergo. One research area where photoabsorption cross-sections play a critical role is in understanding the photochemistry of photolabile volatile organic compounds (VOCs) present in the troposphere. The photolysis rate coefficient, *j*, for a given light-triggered reaction is defined by the following equation^a^
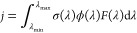
1where σ(λ) is the photoabsorption cross-section of the molecule that will undergo photolysis, ϕ(λ) is the (wavelength-dependent) quantum yield for the photolysis process of interest, and *F*(λ) is the flux of the light source (it being a lamp or the sun). Hence, photoabsorption cross-sections constitute a crucial ingredient to understand and characterize the photochemical reactivities and lifetimes of VOCs upon sunlight absorption. Photoabsorption data for some VOCs are available in online databases such as MPI-Mainz UV/vis Spectral Atlas^1^ and are further used in atmospheric models including the master chemical mechanism to simulate the chemical composition of the atmosphere.^2−5^ Databases of kinetic and photochemical data from IUPAC^6^ and NASA (JPL)^7^ are also available and so are atmospheric models such as TUV.^8^ However, determining the photoabsorption cross-section of transient VOCs experimentally is often hampered by their short-lived nature or difficulties related to their synthesis or isolation. Unknown photoabsorption cross-sections can be estimated using structure–activity relationships (SARs) with known photoabsorption cross-sections of VOCs sharing similar functional groups. Nevertheless, the quantitative precision of this approach is limited due to the possible delocalized nature of molecular excited states, and more general and reliable strategies to determine photoabsorption cross-sections are needed.

Theoretical and computational chemistry could offer an exciting new venue to determine the photoabsorption cross-sections of transient VOCs. Nowadays, molecular photoabsorption spectra can indeed be successfully predicted fully in silico.^9−13^ Theoretically predicted cross-sections are regularly presented alongside experiments, often to interpret electronic characters of absorption bands and their vibronic features. One of the primary issues to address when calculating photoabsorption spectra theoretically is the accurate determination of transition energies and oscillator strengths. Luckily, an important body of work has been dedicated to this issue, benchmarking numerous methods to calculate the excited electronic states of molecules.^14−18^ However, a proper electronic structure method alone is not enough to determine a photoabsorption cross-section, and different strategies have been proposed to reproduce the shape and intensity of absorption bands as detailed below. Determining which of these strategies can be employed to calculate the photoabsorption cross-section of atmospheric VOCs is the central goal of this work.

**Figure 1:**
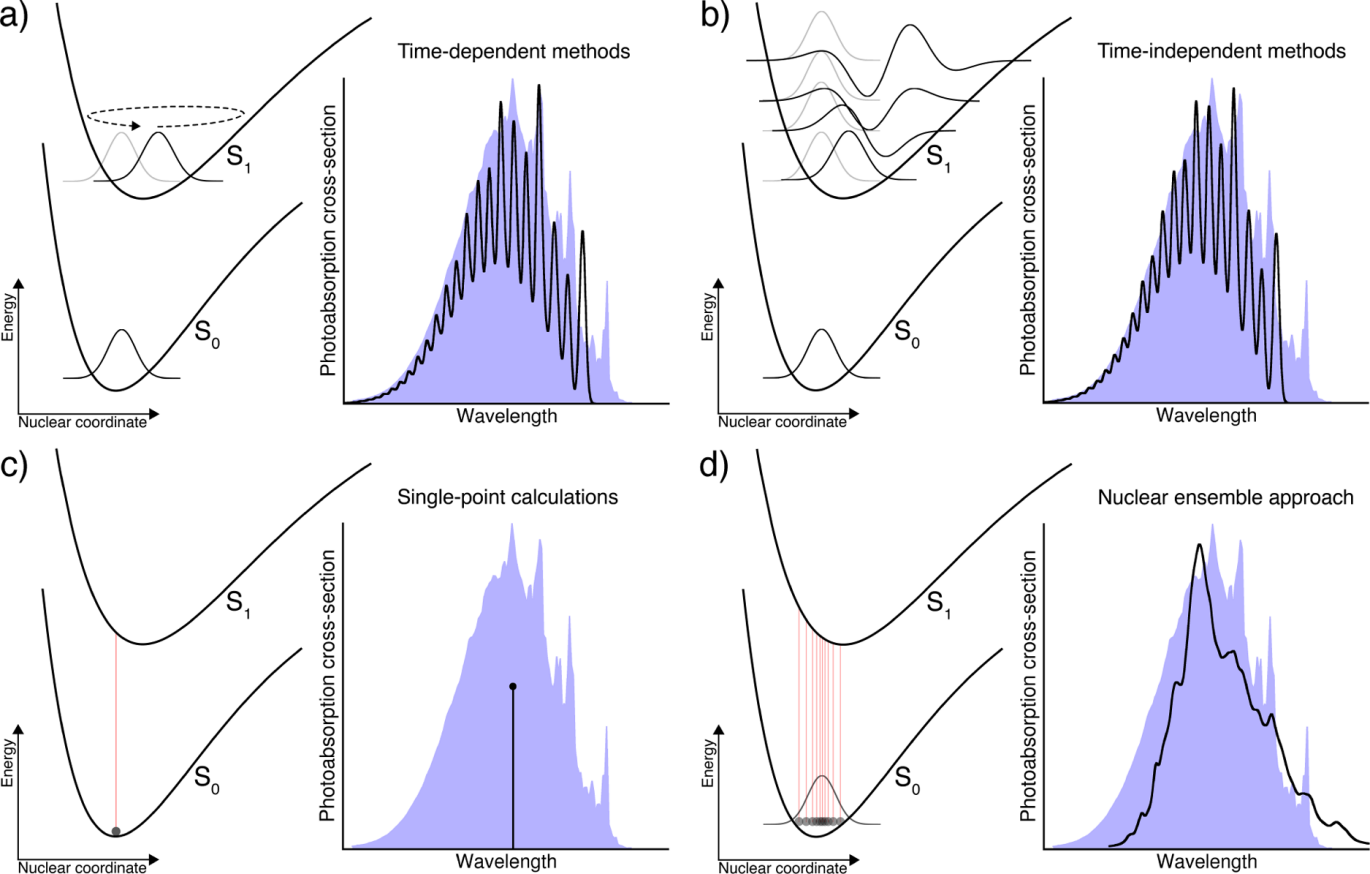
Schematic representation of the different main families of methods to determine photoabsorption cross-sections. For each method, a scheme of the key elements of the simulation is presented on the left, with the potential energy curve for the ground (S_0_) and first excited (S_1_) electronic state. The expected shape of a photoabsorption cross-section is shown on the right (black line), together with a reference experimental spectrum (blue area). (a) Time-dependent methods: the ground-state nuclear wavefunction (black Gaussian in S_0_) is promoted to S_1_ and propagated on this state. (b) Time-independent methods: the overlaps between the initial state of the system (black Gaussian in S_0_ symbolizing the ground electronic and vibrational state of the system) and all the vibrational wavefunctions in S_1_ (black curves in S_1_) are calculated. (c) Single-point calculations: the minimum-energy structure in S_0_ is located (black circle on S_0_), and the vertical excitation energy to S_1_ is computed for this nuclear configuration only (vertical red line between S_0_ and S_1_). (d) NEAs: these methods approximate a quantum distribution representative of the ground-state nuclear wavefunction (gray Gaussian in S_0_). From this distribution, different nuclear configurations are sampled randomly (black dots in S_0_), and for each of them, a vertical transition to S_1_ is calculated (as done in (c)). The resulting spectra are obtained by averaging over all these (broadened) vertical transitions. See the main text for additional information.

There are two fundamentally different ways to approach the theoretical modeling of photoabsorption cross-sections for polyatomic molecules. Some methods employ a time-dependent perspective to the process of light absorption, and some others are formulated in a fully time-independent manner. In the time-dependent approach (Figure 1a), photoabsorption spectra are obtained directly from exact quantum dynamics by the Fourier transform of the nuclear wavepacket autocorrelation function.^19,20^ The process is as follows: one takes the nuclear wavefunction of a molecule in its ground vibrational and electronic state (black Gaussian wavefunction in S_0_ in Figure 1a), excites it vertically to a given excited electronic state (gray Gaussian wavefunction in S_1_ in Figure 1a), and simulates its dynamics (black, evolving nuclear wavefunction in S_1_ in Figure 1a). The interplay between the initial (ground-state) nuclear wavefunction and the nuclear dynamics encodes the vibronic details of a photoabsorption spectrum. Instead of costly full quantum dynamics, various semiclassical approximations can be used to calculate an absorption spectrum.^21,22^ Despite being theoretically rigorous, methods based on quantum wavepacket dynamics can be computationally involved and often require approximations such as reducing the dimensionality of the system to a few important normal modes.

Time-independent approaches based on the calculation of Franck–Condon integrals^23^ between the ground- and excited-state vibronic wavefunctions (black and gray Gaussian in S_1_, Figure 1b) have become a popular way to evaluate vibronically resolved absorption spectra, and this strategy is available in widespread codes such as Gaussian.^24^ Non-Condon effects are often required to account for the dependence of transition dipole moments on nuclear coordinates. To account for such effects, the so-called Herzberg–Teller corrections are typically added to the Franck–Condon terms—a strategy often called Franck–Condon–Herzberg–Teller (FCHT).^25^ The FCHT method usually relies on the harmonic approximation where both the ground and excited electronic states are represented by displaced multidimensional harmonic potentials. Hence, the accuracy of the FCHT method is compromised in molecules with pronounced anharmonicity or dissociative character of the excited states. We note that some FCHT developments propose to include anharmonic corrections^26^ and that a time-dependent formulation of FCHT has also been developed and used.^27^ Including FCHT corrections requires in principle the calculation of vibrational frequencies, both at the ground- and excited-state geometries. Such calculations can be expensive, and several schemes have been proposed to approximate FCHT terms.^25,28^

Vibronic features in photoabsorption spectra are not needed for applications that require only the positions, intensities, and widths of absorption bands, rather than their precise structure. Perhaps, the simplest and most commonly employed strategy in the literature consists of evaluating the excitation energies and oscillator strengths at the optimized ground-state geometry (black circle in S_0_, Figure 1c), producing a stick spectrum (right part of Figure 1c), with transitions that can further be convoluted by Gaussian or Lorentzian functions. While this strategy is easy to deploy, it assumes a symmetric shape for the bands with arbitrarily set phenomenological broadening widths. This method also assumes that the vertical excitation energies match the photoabsorption band maxima. It is however known that band maxima are typically red-shifted with respect to vertical transitions^29^ with shifts as large as 0.4 eV^30^ that are furthermore not necessarily the same for different excited states.

In contrast to the oversimplified method based on a single optimized ground-state minimum structure, the nuclear ensemble approach (NEA)^12^ takes into account molecular vibrations in the ground state. The idea behind the NEA is to sample a large number of molecular geometries (black dots in S_0_, Figure 1d) from the ground-state nuclear density (gray Gaussian in S_0_, Figure 1d). Then, one can compute vertical electronic transitions for each nuclear geometry and convolute the individual transitions by Gaussian or Lorentzian functions. The width of the line shapes is typically chosen to be much narrower than the overall widths of the absorption bands, so that it does not affect the simulated band shapes.^12,31^ As such, the NEA can predict accurate widths, heights, and positions of absorption bands. The NEA accounts for non-Condon effects, as it correctly captures the dependence of transition dipole moments on molecular geometry. On the other hand, it completely lacks the description of vibronic structures, as no information about the nuclear wavefunction(s) in the excited states is required (right part of Figure 1d). We note that a formal derivation and justification of the NEA were presented in earlier studies.^12,32^ Similar approaches have been proposed already in 1980s under the name of “reflection principle”.^33,34^ The method has been successfully employed for a broad variety of molecular systems (e.g., refs (35−44)), and an optimal sampling strategy from a statistical perspective has been discussed recently.^45^

One key question remains with the NEA: how can we obtain the best-possible approximation for the ground-state nuclear distribution used for the sampling of geometries? A system in thermal equilibrium can be conveniently sampled from a long Born–Oppenheimer ground-state dynamics within a canonical ensemble. However, thermal (i.e., Boltzmann) sampling at room temperature does not recover the kinetic energy (and potential energy) of the zero-point vibrational motion.^46^ As a result, the NEA photoabsorption spectra obtained from a thermal distribution may lead to bands with qualitatively wrong widths.^46^ One way to obtain a quantum distribution for the ground vibrational state of a molecular system is to use a Wigner distribution. A Wigner distribution offers a way to map quantum nuclear densities to a sort of classical phase space, and its combination with the NEA provides superior photoabsorption cross-sections when compared to thermal sampling.^46^ Temperature effects can also be straightforwardly included within Wigner sampling (in a harmonic case).^12^ However, obtaining an exact Wigner transform of quantum wavefunctions is very challenging for anharmonic multidimensional molecules. In practice, one often has recourse to an approximate Wigner distribution evaluated within the harmonic approximation, formed from the uncoupled normal modes of the ground-state potential energy surface. While Wigner sampling is a conceptually simple black-box procedure that only requires the calculation of normal modes at the ground-state minimum geometry, its underlying approximations—the use of harmonic, uncoupled modes—may limit its performance for molecules like VOCs. From this perspective, sampling the molecular geometries from molecular dynamics still appears to be more beneficial for anharmonic, large, and flexible molecular systems.

A possible strategy to include nuclear quantum effects in molecular dynamics simulations is to rely on a path integral representation of the partition function—a method called path integral molecular dynamics (PIMD).^47^ Since PIMD simulations are often computationally expensive, an alternative approximate method called quantum thermostat (QT) sampling was introduced by Ceriotti et al. to obtain a quantum nuclear density.^48,49^ In this approach, a classical non-equilibrium dynamics is performed with a generalized Langevin equation (GLE) thermostat that maintains different normal modes at different frequency-dependent temperatures. The thermostat parameters are fitted so that the resulting density distributions match the distributions of a quantum harmonic oscillator. Note, however, that the dynamics itself does not employ a harmonic approximation. QT simulations are exact for a system of uncoupled harmonic oscillators but also perform well for anharmonic molecules. It can accurately sample both high-frequency modes, where quantum effects dominate, and low-frequency modes, where anharmonic and temperature effects are more important.^50^ Nevertheless, care should be taken when applying QT sampling for strongly anharmonic systems, such as weakly bonded complexes, or in the low-temperature regime.^48^ In these cases, one can employ other strategies such as PI+GLE^51^ or PIGLET,^52^ where the idea of QT is combined with PIMD.^53^

A few earlier studies made use of the NEA using an approximate Wigner distribution or PIMD distribution to calculate in silico the photoabsorption cross-section of atmospheric VOCs.^36,37,54−57^ In this work, we wish to evaluate the performance of the NEA when combined with Wigner or QT sampling to predict the photoabsorption cross-sections of four exemplary oxygenated VOC molecules of great interest in atmospheric photochemistry: acrolein, methylhydroperoxide, 2-hydroperoxy-propanal (2-HPP), and pyruvic acid. We focus our attention on (i) the influence of the sampling on the result of the NEA for the different VOCs, (ii) the limitation of Wigner sampling for flexible VOCs or microsolvated VOCs, and (iii) the comparison of photoabsorption cross-sections obtained in silico and via SARs for a multichromophoric VOC.

## 2 Computational Details

### 2.1 Electronic Structure

Except where stated otherwise, we employed the same level of theory for all molecules, and all electronic-structure calculations were performed with Gaussian 09 revision D.01.^24^ Ground-state (S_0_) minima and vibrational frequencies were obtained with density functional theory (DFT) using the PBE0 functional^58^ and the 6-311G* basis set. The same level of theory was utilized for the ab initio MD simulations using the QT approach as detailed below.

Excited-state energies and oscillator strengths were calculated with linear-response time-dependent DFT (LR-TDDFT)^59,60^ using the Tamm–Dancoff approximation (TDA),^61^ at the PBE0/6-311G* level. For methylhydroperoxide, the 6-311+G* basis set was employed since diffuse basis functions are needed to describe the valence-Rydberg mixing in peroxide excited states.^54^

As this work focuses on the sampling of ground-state geometries, we do not provide a detailed comparison of different electronic structure methods. We refer the interested reader to the abundant literature on this subject.^14−16^

### 2.2 Ground-State Sampling

Wigner sampling of the vibrational ground state within the harmonic approximation was used as implemented in the Newton-X 2.0 package.^62^

Molecular dynamics with the QT was performed with the ABIN code,^63^ coupled to the TeraChem v1.9 package for the electronic structure,^64,65^ using the same ground-state electronic structure level as elsewhere (PBE0/6-311G*). Parameters for the thermostat were taken from the GLE4MD webpage,^66^ using a target temperature of *T* = 296 K and parameters *N*_s_ = 6, ℏω/*kT* = 20, and strong coupling. The molecular dynamics time step was ∼0.5 fs. The equilibration time was determined by monitoring the convergence of the average kinetic energy temperature. For molecules with multiple conformers, independent trajectories starting from a given minimum geometry were recorded for each conformer. Conversions between conformers were only observed in the simulations of 2-HPP and taken into account for the overall sampling (see below).

### 2.3 Spectral Calculations

Photoabsorption cross-sections from the NEA were computed with the Newton-X 2.0 package^12,62^ interfaced to Gaussian 09 revision D.01^24^ using the equation^31^
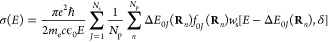
2where *E* is the photon energy, *e* is the electron charge, *m*_e_ is the electron mass, ϵ_0_ is the vacuum permittivity, and *c* is the speed of light. Δ*E*_0*J*_(**R**_*n*_) corresponds to the vertical excitation energy between the ground state 0 and the excited electronic state *J*, for the *n*th sampled molecular geometry with the corresponding nuclear configuration **R**_*n*_. *f*_0*J*_(**R**_*n*_) is the corresponding oscillator strength at nuclear configuration **R**_*n*_. *w*_s_[*E* – Δ*E*_0*J*_(**R**_*n*_), δ] is a normalized line shape centered at energy Δ*E*_0*J*_(**R**_*n*_) and with a phenomenological width δ. In our calculations, we used a Lorentzian shape for *w*_s_ with a phenomenological broadening of 0.05 eV (default in Newton-X, discussed in ref (12)). The first sum runs over *N*_s_ electronic states. The second sum runs over all the *N*_p_-sampled nuclear geometries. *N*_p_ is set to 500 for both Wigner and QT sampling, for each conformer.

The overall spectra were evaluated by scaling the contributions of each conformer by their Boltzmann factors—two conformers for acrolein, one conformer for methylhydroperoxide, eight conformers for 2-HPP, two conformers for pyruvic acid in the gas phase, and one conformer for pyruvic acid with a single water molecule. For each molecule, the calculated spectra account solely for the S_1_ ← S_0_ transition, which is well separated from the other transitions at higher energy, except for methylhydroperoxide where the lowest five singlet excited states need to be considered.

For acrolein, the vibrational frequencies were in addition evaluated at the minimum-energy geometry of the lowest singlet excited state (S_1_) to calculate FCHT with an adiabatic Hessian (FCHT-AH) photoabsorption cross-section. Calculations were performed using DFT/PBE0/6-311G* and LR-TDDFT/TDA/PBE0/6-311G*. The lines obtained from the FCHT-AH calculations were broadened by Gaussian functions with a half-width at half-maximum set to ∼0.019 eV. For reasons detailed in the respective result sections, we did not calculate the FCHT spectra for the other molecules due to the presence of multiple conformers and/or dissociative excited states.

We note that no shifts or scaling factors were applied on the presented photoabsorption cross-sections.

## 3 Results and Discussion

### 3.1 Acrolein—A Harmonic Molecule

Acrolein is a simple unsaturated carbonyl, which undergoes a range of atmospheric oxidation reactions taking place both in the dark and in the presence of sunlight.^67^ The UV/vis absorption properties of acrolein have been investigated both spectroscopically^68−71^ and theoretically.^13,72−74^ Acrolein absorbs very weakly in the actinic region due to the nπ* nature of its low-lying singlet excited state.^72^ Excited states with an nπ* character typically have small oscillator strengths due to the mutual orthogonality of hole (n) and particle (π*) orbitals,^75^ and the inclusion of non-Condon effects becomes important to reveal the proper intensity of nπ* bands.

**Figure 2:**
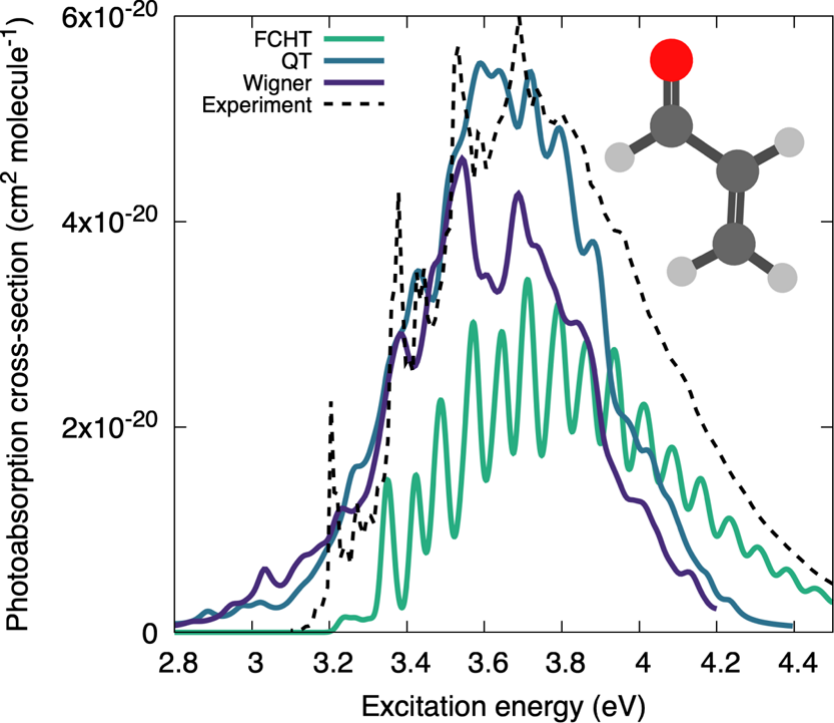
Calculated and experimental photoabsorption cross-sections of acrolein. The experimental spectrum (Magneron, 1998, unpublished) was obtained from the MPI-Mainz UV/vis Spectral Atlas.^1^

The comparison between the available experimental data^1^ and predictions from FCHT-AH and the NEA is shown in Figure 2. The experimental spectrum exhibits a clear vibronic structure, especially in the low-energy region, and is highly asymmetric with a pronounced high-energy tail. The presence of this vibronic progression can be attributed to the bound nature of the nπ* (S_1_) excited state. The FCHT spectrum reproduces all the general features of the experimental spectrum and in particular shows that the high-energy tail can be attributed to the vibronic progression. Neglecting the HT contribution leads, as expected, to a smaller cross-section, and the use of the vertical-gradient (VG) approximation for FCHT (FCHT-VG) still allows us to obtain a photoabsorption cross-section in good agreement with experiment (see Figure S1 in the Supporting Information). The smaller height of the FCHT-AH spectrum in comparison to the experimental one can be attributed to the level of electronic structure theory. Using a larger basis set further improves the agreement with experiment (see Figure S2 in the Supporting Information).

In contrast to the FCHT-AH method, the NEA is incapable by construction of describing such vibronic structures—the features appearing in the NEA spectra are due to a finite number of geometries in the sampling. Nevertheless, the NEA using both Wigner and/or QT for the sampling of the ground-state nuclear density offers a good match with the experimental spectrum, in particular for the absolute cross-sections with the QT sampling.

Overall, the three different theoretical approaches based on a common level for the electronic structure provide a reliable estimate of the photoabsorption cross-section with a reasonable agreement with the experimental data. This agreement is mainly due to the fact that acrolein follows closely the harmonic approximation in its ground and first excited state—a critical feature for the success of the FCHT and NEA/Wigner as detailed further later.

### 3.2 Methylhydroperoxide—Limitations of the Harmonic Approximation

Our second example focuses on methylhydroperoxide, the simplest organic hydroperoxide. Peroxides are atmospherically relevant as side products in combustion chemistry and play an important role in the formation of secondary organic aerosols.^76,77^ In the atmosphere, they are formed as termination products of VOC degradation under low-NOx conditions (RO_2_ + HO_2_ reactions).^77^ Alkyl peroxides undergo photolysis which involves dissociation of OH, H, and O radicals.^54,78^ This is due to the dissociative nature of their low-lying excited states, primarily of nσ* character, where the σ* orbital is antibonding with respect to the O–O or O–H bond.^54^ The dissociative nature of the excited states excludes the applicability of the FCHT method for the absorption spectrum—excited states do not possess a minimum but undergo a barrierless stabilization along the dissociative pathway. This obstacle is not shared by the NEA, which can be applied also in the case of unbound excited states. The NEA is also a more elegant approach when a manifold of multiple electronic states contributes to the overall absorption cross-section, which is the case in peroxides.

**Figure 3:**
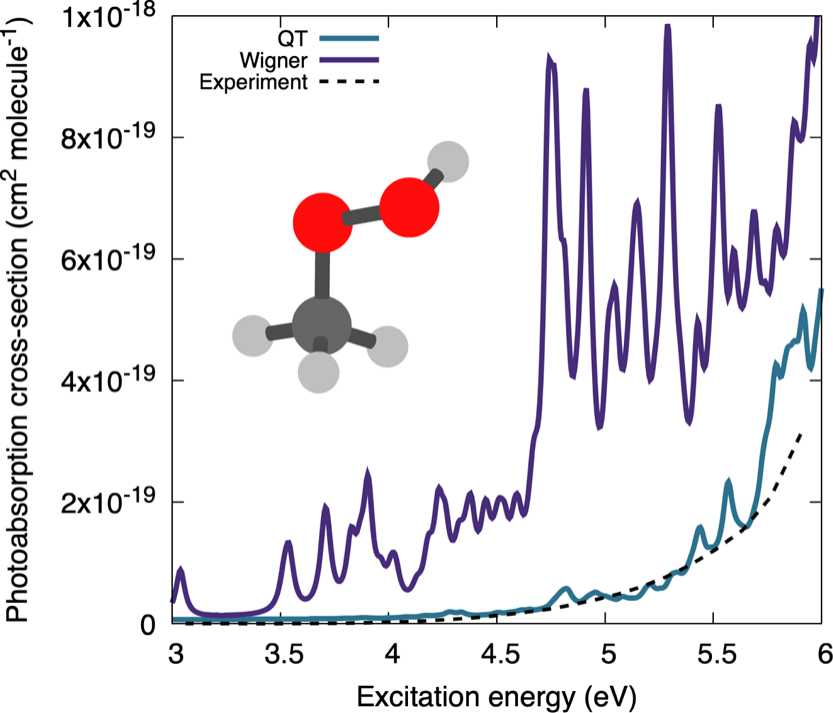
Calculated and experimental photoabsorption cross-sections of methylhydroperoxide. The experimental spectrum was obtained by combining data from refs (79) and (80), as recommended in the MPI-Mainz UV/vis Spectral Atlas.^1^

Figure 3 offers a comparison between the photoabsorption cross-sections of methylhydroperoxide predicted by the NEA, with both Wigner and QT sampling, and the experimental reference. Unlike the case of acrolein, the NEA results based on a Wigner or QT sampling show noticeable differences. Only the cross-section obtained from a QT sampling exhibits a good agreement with experimental data. The NEA using Wigner sampling predicts transitions with a higher oscillator strength in the low-energy range of the spectrum, appearing as sharp features on the photoabsorption cross-section. While such sharp features could be averaged out in the NEA spectrum by increasing the number of sampled geometries or adjusting the line broadening, the problem would persist—the absorption intensity would simply be too high in comparison to the experimental spectrum.

**Figure 4:**
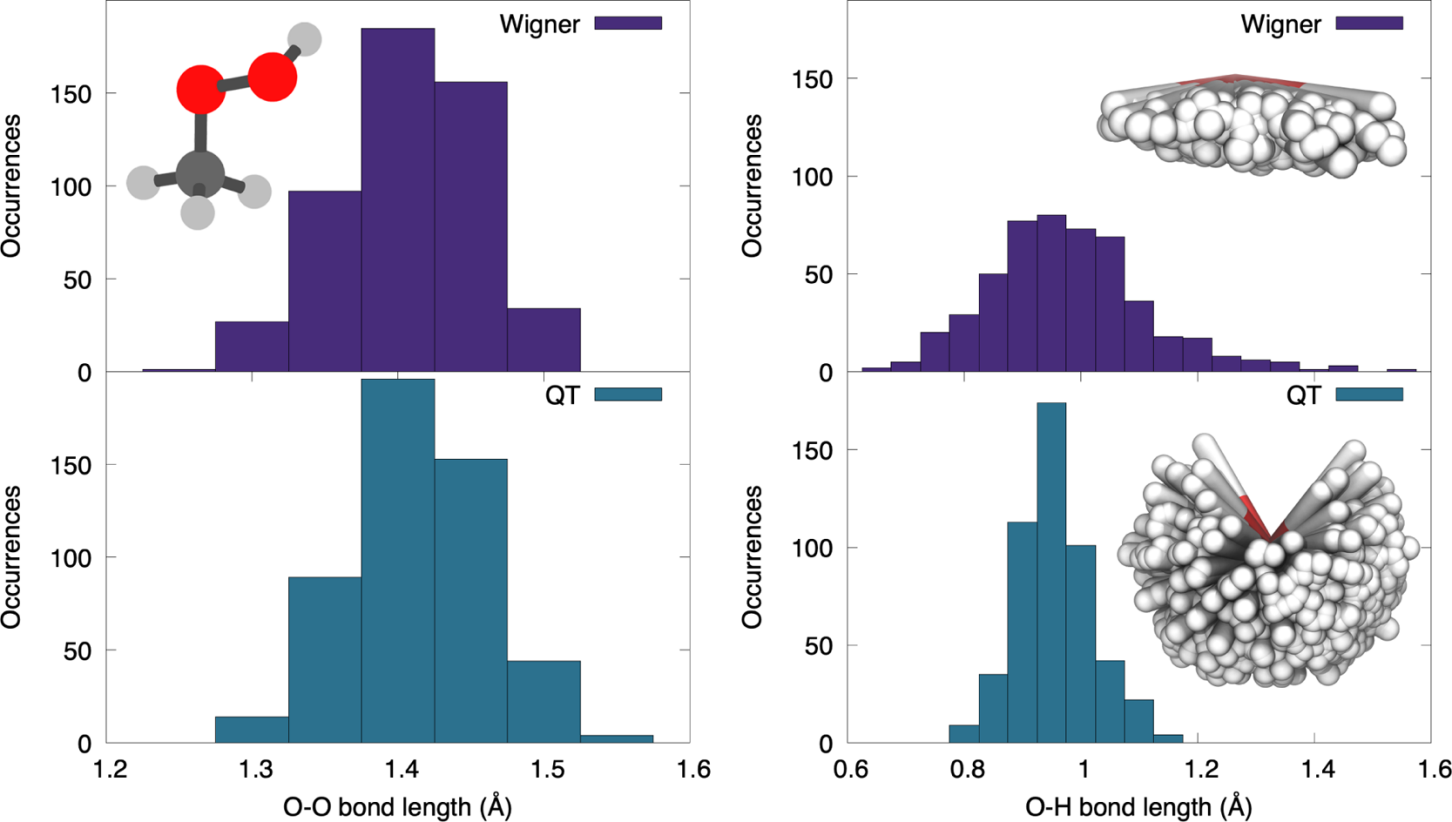
Distribution of O–O (left) and O–H bond lengths (right) in methylhydroperoxide for a sampling obtained from a Wigner distribution (upper panels) or QT dynamics (lower panels). The insets show the spatial distribution of hydrogen atoms from the OH group in the 500 geometries obtained from the two types of sampling.

The issues experienced by Wigner sampling for methylhydroperoxide are disclosed by analyzing the distributions of bond lengths. Figure 4 shows histograms of O–O and O–H bond lengths in the Wigner and QT sampling. The distributions of O–O bond lengths are very similar with the two types of sampling, as it might be expected from a rather harmonic bond. The Wigner and QT distributions are however surprisingly different when it comes to the O–H bond (right panel of Figure 4). The QT sampling predicts O–H bond lengths to vary between 0.8 and 1.2 Å, while the distribution is much broader with Wigner sampling, extending up to 1.6 Å. Wigner sampling in its practical implementations for molecules—describing a molecule as a set of uncoupled harmonic oscillators using normal modes described in Cartesian coordinates—has an inherent problem with sampling soft low-frequency modes.^81,82^ In particular, rectilinear normal modes used for the Wigner sampling constitute a bad representation for torsions, as atoms are displaced along the normal-mode vectors, that is, along straight lines, being sheared instead of rotated.^50,81,83,84^ For light atoms such as hydrogen, this causes too large interatomic displacements. In the particular case of methylhydroperoxide, the O–H stretching mode itself is well described, but the issue emerges when the low-energy −C–O–O–H torsional mode is sampled. The poor sampling of this torsional mode leads to an artificial stretching of the O–H bond with no energy penalty.

How does this biased O–H distribution end up affecting the photoabsorption cross-section then? Looking back at the results presented in Figure 3, we can analyze the electronic characters of the transitions resulting in the peaks with a high oscillator strength in the NEA/Wigner spectrum. This analysis reveals that these high peaks correspond to transitions with an nσ* character, where the σ* orbital is antibonding with respect to the O–H bond. This character corresponds to an S_2_ ← S_0_ transition at the minimum-energy geometry, but the energy of this transition decreases sharply when the O–H bond is elongated. In addition, the nσ*(O–H) transition is characterized by a larger oscillator strength than the nσ*(O–O) one. Hence, the artificial elongation of the O–H bond caused by the Wigner sampling leads to the appearance of artificial high intensity of the nσ*(O–H) transitions in the lower spectral range that pollute the overall cross-section. The QT removes this artifact as the dynamics per se does naturally account for the couplings between the different normal modes, proving a more reliable sampling procedure for this molecule. To confirm the influence of the −C–O–O–H torsional mode in the NEA spectrum, we removed this low-frequency mode before performing the Wigner sampling. The resulting O–H bond length distribution shows a similar width as that obtained with QT, and the corresponding NEA/Wigner spectrum agrees well with the NEA/QT results and the experimental cross-section (see the Supporting Information, Figure S3).

### 3.3 2-Hydroperoxy-propanal—on the Use of SARs for Photoabsorption Cross-Sections

**Figure 5:**
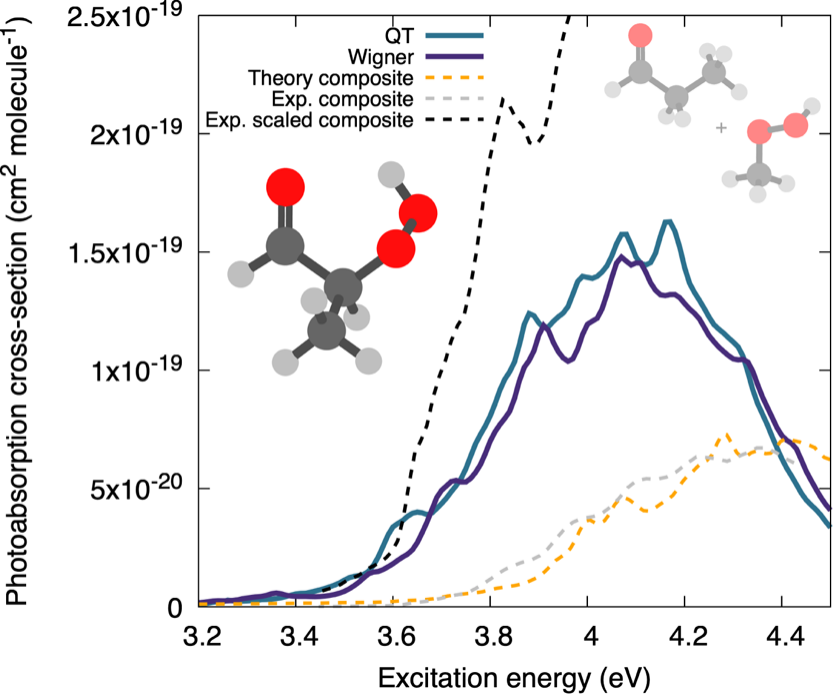
Calculated and experimental photoabsorption cross-sections of 2-HPP. The theoretical composite spectrum was obtained by combining the calculated photoabsorption cross-sections of methylhydroperoxide and propanal, depicted on the right inset (see text for additional details). The experimental composite spectra were digitized from ref (87).

Unlike acrolein and methylhydroperoxide, the photophysics and photochemistry of most atmospheric VOCs have not been fully characterized experimentally. For such compounds, other strategies were proposed to estimate their photoabsorption cross-sections such as SARs, where molecular properties are inferred based on chemically similar compounds for which accurate measurements are available. For instance, the photoabsorption cross-section of a multifunctional molecule may be estimated based on smaller monofunctional fragments, each containing one of the functional groups of the parent multifunctional molecule (for example, see refs (85) and (86)). Peeters et al. used this strategy in their study of photolysis of α-hydroperoxy-carbonyls—molecules that contain both a peroxide and carbonyl moiety.^87^ The composite photoabsorption cross-section of 2-HPP (left structure in Figure 5) was estimated from the sum of photoabsorption cross-sections of methylhydroperoxide and propanal (right structures of Figure 5). Due to possible intramolecular interactions (e.g., H-bonding) between the two functional groups in 2-HPP, the total composite photoabsorption cross-section was multiplied with an enhancement factor that was estimated from the data of chemically similar β-hydroperoxy-carbonyls.^87^

The experimental composite and scaled composite spectra of 2-HPP are shown in Figure 5 (dashed lines). To validate our approach, we first reproduced the unscaled experimental composite spectra fully by theory, combining the NEA photoabsorption cross-sections of propanal and methylhydroperoxide (as calculated in Figure 3). For methylhydroperoxide, we took our best results based on QT sampling, while the propanal cross-section was calculated using Wigner sampling (given the overall good agreement between Wigner and QT for acrolein). Overall, the theoretical and experimental composite spectra agree very closely. However, the theoretical approaches described in this work allow us to study the original 2-HPP molecule itself, bypassing the need for the use of SARs. The photoabsorption cross-section of 2-HPP was calculated with the NEA using two sampling procedures—Wigner and QT—accounting for the eight low-energy conformers of the molecule. Note that the flexibility of 2-HPP and its numerous conformers in the ground and excited (S_1_) state^87^ complicates the use of the FCHT method—the harmonic approximation not being reliable for several of the nuclear wavefunction overlaps calculated; we omitted the results of this approach. The NEA results for QT or Wigner sampling (Figure 5) are in good agreement in this example. More importantly, these results clearly indicate that there is a sizable increase in absorption intensity as compared to the theoretical composite spectra at this level of electronic-structure theory, but the enhancement is significantly smaller than the one predicted by scaling reported in earlier work.^87^ By analyzing the photoabsorption cross-section for the main conformers (Figure S4 in the Supporting Information), it appears that conformers displaying an intramolecular H-bond between the carbonyl group and the hydrogen of the peroxide moiety have a larger photoabsorption cross-section than the others. This shows that using SARs bears a risk of over- or underestimating the photoabsorption cross-sections, but this risk can be assessed using the theoretical approaches described here.

### 3.4 Pyruvic Acid—Microsolvation Effects

Pyruvic acid is an abundant atmospheric compound originating from the oxidation of isoprene and a proxy for α-dicarbonyls.^88^ The photochemistry of pyruvic acid and its water clusters has been studied extensively both by theory^89−91^ and experiments.^88,92−94^ A UV spectrum is available for the pristine pyruvic acid molecule,^95^ while pyruvic acid–water complexes are harder to characterize due to keto–enol tautomerization where different species can be found in equilibrium.^89^ Since our goal is not to study the complexity of aqueous pyruvic acid photochemistry, we focus here on a simple monohydrate of pyruvic acid, where the carbonyl and carboxyl groups are H-bonded to a single water molecule.

**Figure 6:**
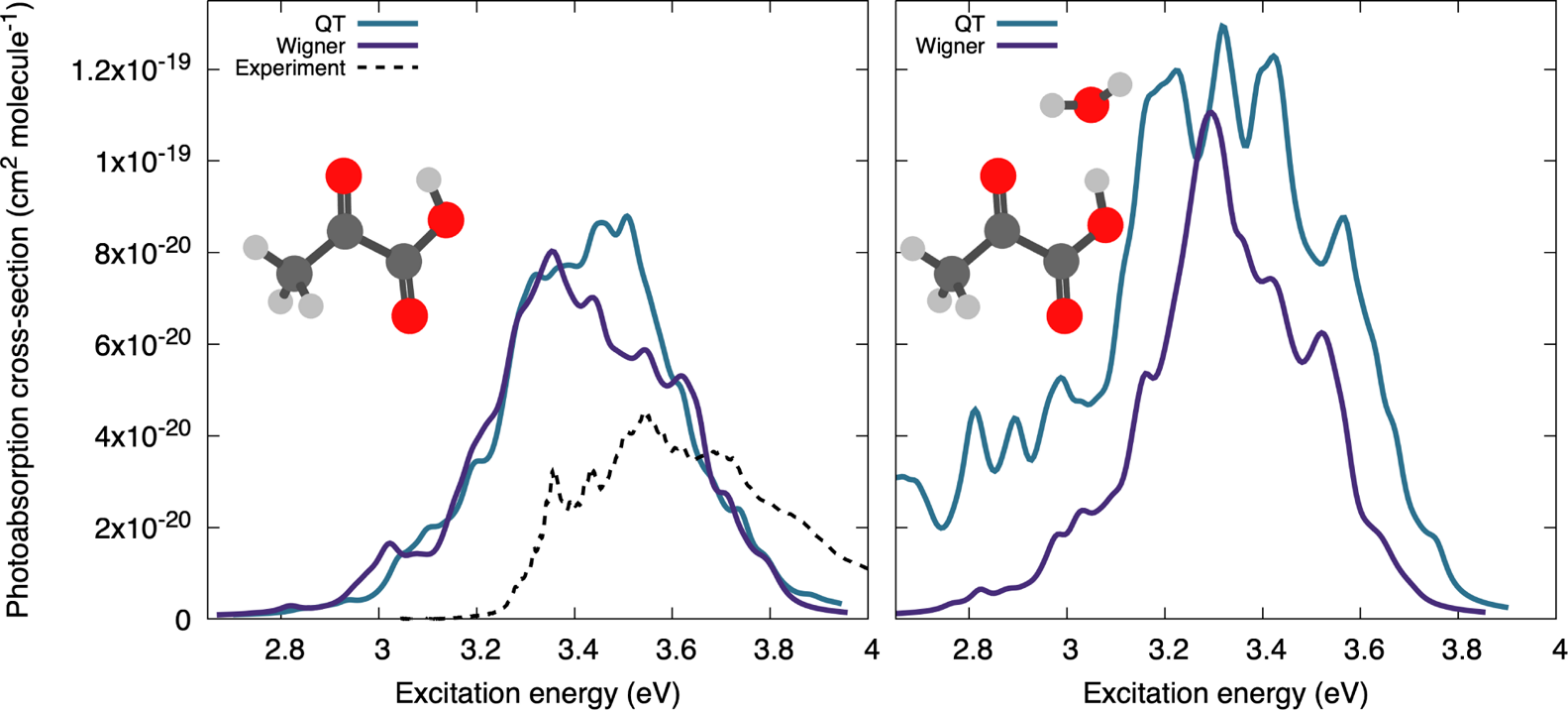
Calculated and experimental photoabsorption cross-sections of pyruvic acid (left) and its monohydrate (right). Experimental data for pyruvic acid were obtained from ref (95).

Figure 6 shows the photoabsorption cross-sections of pyruvic acid (left panel) and its monohydrate (right panel). The theoretically predicted NEA cross-sections of pyruvic acid (using QT and Wigner for the sampling) in vacuo are both slightly red-shifted and enhanced as compared to the experimental data, although the widths of the bands are similar. However, the discrepancy in the position and intensity of the absorption peak is mostly due to the level of the electronic structure employed—using a correlated wavefunction-based method instead of LR-TDDFT significantly improves the agreement with experiment (see Figure S5 in the Supporting Information).

**Figure 7:**
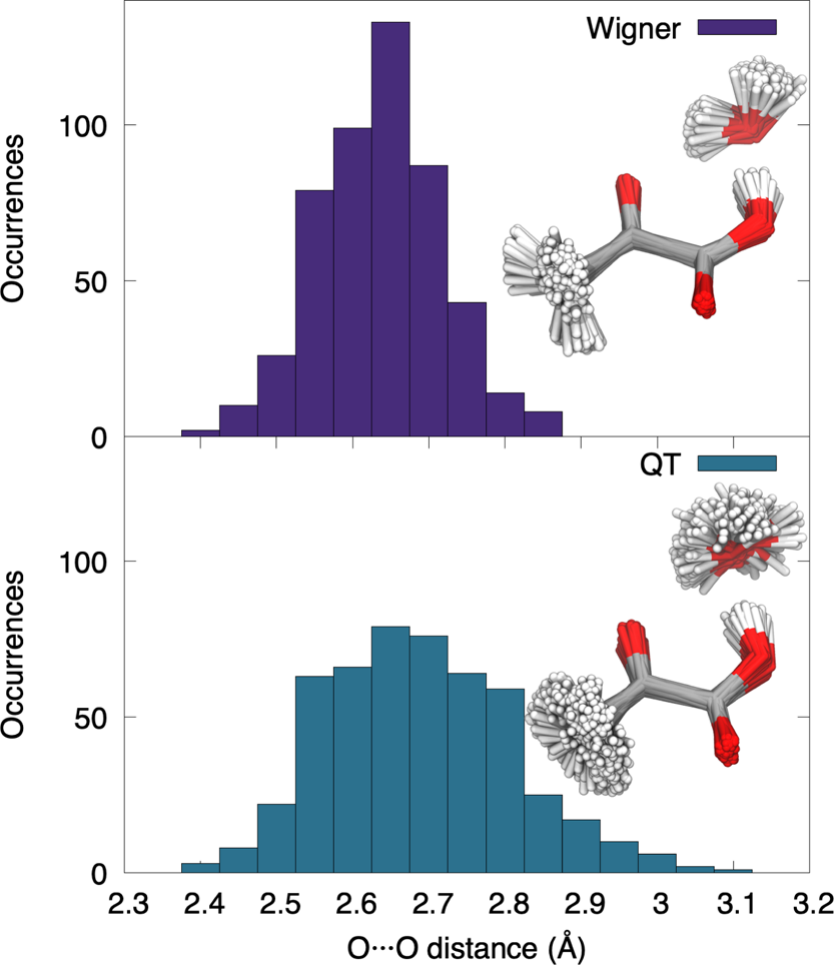
Distribution of O···O interatomic distances between water and carbonyl oxygens in pyruvic acid monohydrate for a Wigner distribution (upper panel) and QT sampling (lower panel). Insets show the overlapping 500 molecular geometries from the two types of sampling.

While QT and Wigner samplings predict very similar results for the pristine molecule, the two sampling methods exhibit much larger discrepancies when a water molecule is H-bonded to the pyruvic acid (right panel of Figure 6). The NEA photoabsorption cross-section based on a QT sampling is significantly broader than the one obtained from a Wigner distribution. In fact, the Wigner sampling does not show any significant broadening with respect to pyruvic acid alone (compare the solid violet curves in both panels of Figure 6), although a peak broadening due to solvation is a well-known phenomenon in absorption spectroscopy.^96^ The origin of this broadening is again disclosed by analyzing the distributions of interatomic distances. As shown in Figure 7, Wigner sampling predicts a too narrow distribution of the distances between the water oxygen and the carbonyl oxygen. This distance is chosen because the main absorption band in the actinic region originates from an nπ* transition that is primarily localized on the carbonyl moiety. The Wigner distribution is based on harmonic displacements around the (S_0_) minimum-energy geometry, which prevents a proper description of the flexibility of the hydrogen-bonded water molecule (see insets of Figure 7). The QT sampling accounts for this flexibility and its influence on the carbonyl chromophore. As a result, the NEA/QT absorption band of the microsolvated pyruvic acid is broadened with respect to the pure molecule.

## 4 Conclusions

In this work, we investigated the use of simple computational methodologies for predicting the photoabsorption cross-sections of VOCs. Our findings indicate that the FCHT technique is a valuable tool to predict the photoabsorption cross-section of atmospheric organic molecules if such molecules are rather rigid (i.e., they fulfil the underlying harmonic approximation of FCHT calculations) and exhibit bound excited electronic states. For molecules not following this prescription, the NEA constitutes an excellent alternative to FCHT, as it can straightforwardly treat flexible molecules with a large number of electronic transitions, including unbound ones, even if this strategy lacks a description of vibronic transitions in the resulting photoabsorption cross-sections. The NEA can predict correct widths, heights, and positions of absorption bands, which is sufficient for most atmospheric applications, such as the evaluation of photolysis rate coefficients. The NEA might strongly complement the approximate SAR approaches that are often used to predict photoabsorption cross-sections of transient and multifunctional atmospheric molecules, as highlighted with the example of 2-HPP.

However, one should be extremely cautious about the sampling of ensemble configurations when using the NEA. Earlier studies clearly identified the limitation of using a classical thermal sampling, as it does not properly map the nuclear phase space of a quantum system due to the too small internal energies deposited in vibrational degrees of freedom.^46^ Building upon this knowledge, we investigated and compared here the performance of two quantum sampling approaches for VOCs—Wigner and QT. The NEA is most commonly used with Wigner sampling, which properly treats zero-point vibrational effects in the ground state of a quantum system. Wigner sampling often performs sufficiently well, and it can be calculated in a black-box manner from harmonic vibrational modes. However, we showed that QT sampling appears superior to the simple Wigner distribution in situations when the latter is inherently limited by its ground-state harmonic approximation, that is, whenever low-frequency anharmonic modes may affect the computed photoabsorption cross-sections. Such situations are not uncommon, as shown in this work for the examples of methylhydroperoxide and microsolvated pyruvic acid. Despite this advantage, QT sampling is significantly more computationally expensive than using the simple Wigner approach. Wigner sampling only requires locating minima on the ground-state potential energy surface and calculating for each of them the corresponding vibrational frequencies. QT requires the use of ab initio molecular dynamics, which, in the particular case of methylhydroperoxide presented in this work, required the evaluation of ∼97 000 (ground-state) electronic energies and nuclear gradients.

An easy fix to the issue experienced by the NEA/Wigner with low-frequency modes is to filter out possibly problematic modes with a vibrational frequency of only a few hundreds of cm^–1^ when generating the Wigner distribution^97,98^—such a strategy appeared to fix the artifacts observed in the photoabsorption cross-section of methylhydroperoxide.

Overall, we believe that the NEA combined with an adequate sampling strategy—and a proper level of electronic structure theory—can offer a powerful tool for the determination of unknown photoabsorption cross-sections for transient VOCs in atmospheric chemistry. This work also served as a stepping stone for the current development of an automated web server for atmospheric modelers that will generate photoabsorption cross-sections with minimal user input using the NEA.

## Supporting Information Available

Comparison of FCHT-AH, FCHT-VG, and FC-AH spectra for acrolein, FCHT-AH spectrum with a larger basis set, NEA/Wigner photoabsorption cross-section for methylhydroperoxide without sampling of the −C–O–O–H torsion mode, and photoabsorption cross-sections for dominant conformers of 2-HPP, NEA/Wigner photoabsorption cross-section of pyruvic acid with SCS-ADC(2)/cc-pVTZ (PDF)

## Footnotes

a Note that eq 1 is also sometimes written with dependencies on the solar zenith angle, temperature, and pressure.
