# Supplementary material for: Calculating Photoabsorption Cross-Sections for Atmospheric Volatile Organic Compounds
Source: ACS Earth Space Chem. 2021 Dec 17;6(1):207–17. doi: 10.1021/acsearthspacechem.1c00355 (PMC8785186; doi:10.1021/acsearthspacechem.1c00355)
Supplement: Supplementary file 1 — sp1c00355_si_001.pdf [file sp1c00355_si_001.pdf]

Supporting Information for: Calculating Photoabsorption Cross-Sections for Atmospheric Volatile Organic Compounds

Antonio Prlj,[†] Emanuele Marsili,[†] Lewis Hutton,[†] Daniel Hollas,[†,‡] Darya Shchepanovska,[¶] David R. Glowacki,[§,‖] Petr Slavíček,[‡] and Basile F. E. Curchod[*,†]

†*Department of Chemistry, Durham University, Durham DH1 3LE, United Kingdom*

‡*Department of Physical Chemistry, University of Chemistry and Technology, Prague, Technická 5, 16628 Prague, Czech Republic*

¶*Centre for Computational Chemistry, School of Chemistry, University of Bristol, Bristol BS8 1TH, UK*

§*ArtSci International Foundation, 5th floor Mariner House, Bristol BS1 4QD, UK*

‖*CiTIUS Intelligent Technologies Research Centre, Rúa de Jenaro de la Fuente, s/n, 15705 Santiago de Compostela, A Coruña, Spain*

E-mail: basile.f.curchod@durham.ac.uk

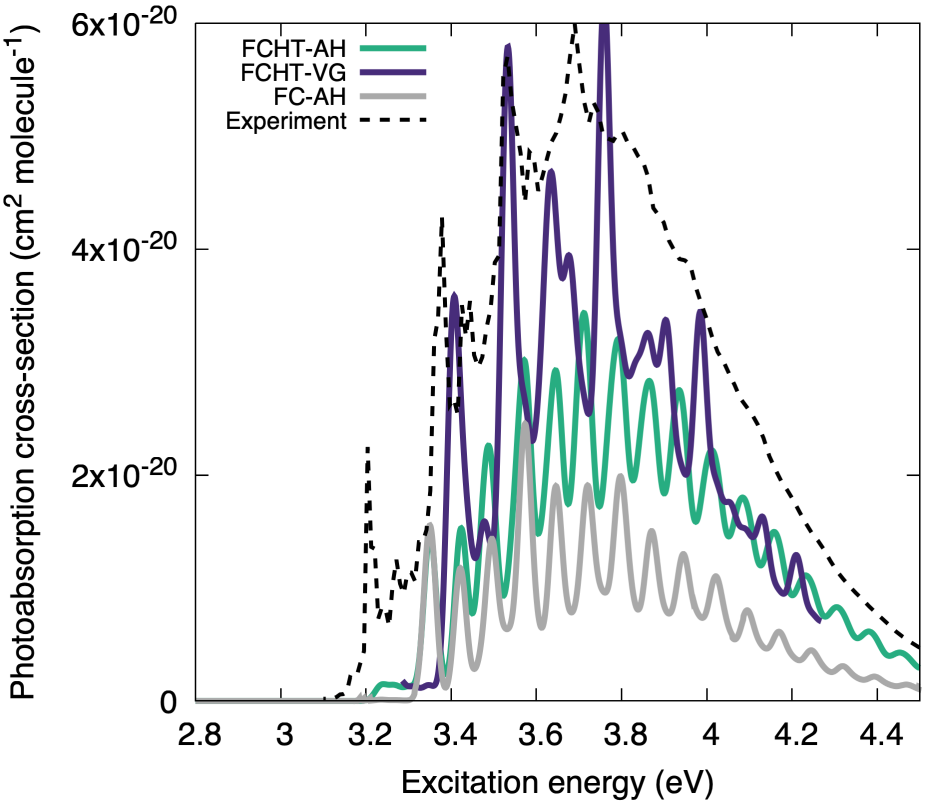

Figure S1: Comparison between the Adiabatic Hessian (AH) and Vertical Gradient (VG) version of the FCHT approach for the photoabsorption cross-section of acrolein. The Franck-Condon photoabsorption cross-section (with the AH) is also displayed and shows the contribution of the HT terms to the intensity of this band. The experimental spectrum (Magneron, 1998, unpublished) was obtained from the MPI-Mainz UV/Vis Spectral Atlas.[1]

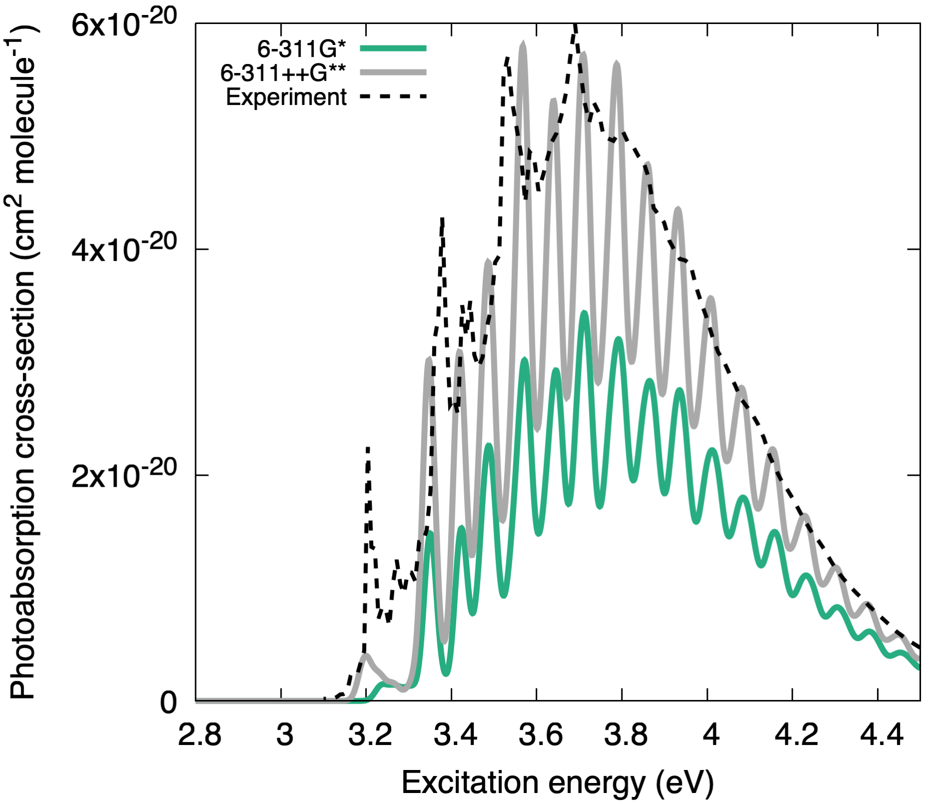

Figure S2: Influence of the basis set on the FCHT photoabsorption cross-section of acrolein. The experimental spectrum (Magneron, 1998, unpublished) was obtained from the MPI-Mainz UV/Vis Spectral Atlas.[1]

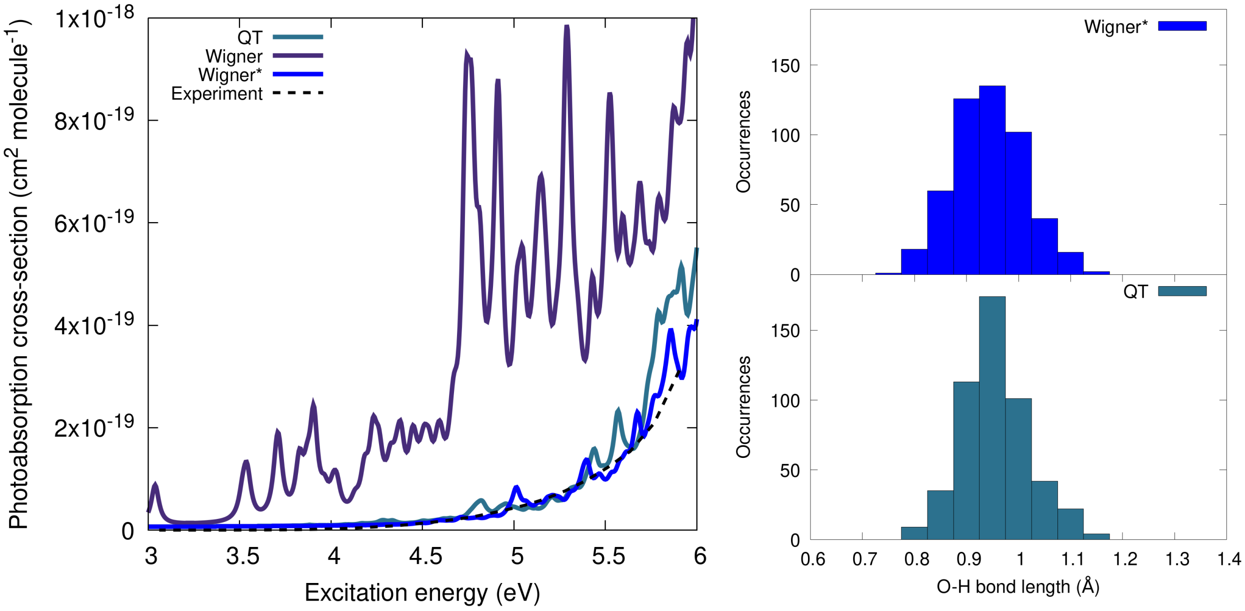

Figure S3: Left panel: Calculated and experimental photoabsorption cross-sections of methylhydroperoxide. Wigner* corresponds to Wigner sampling where the lowest frequency normal mode ($183.7\,\mathrm{cm}^{-1}$) was discarded. Right panel: Distribution of O−H bond lengths in methylhydroperoxide for the sampling obtained from the Wigner* distribution and the QT dynamics.

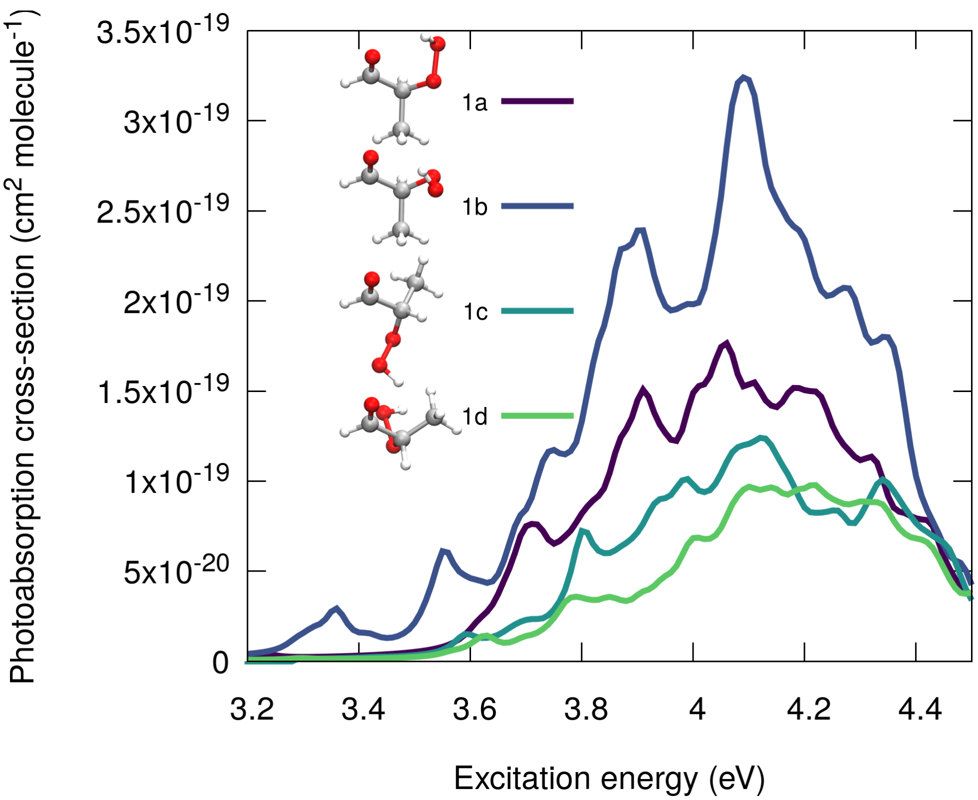

Figure S4: Calculated photoabsorption cross-sections of 2-HPP conformers exhibiting different intramolecular interactions. Conformers 1a and 1b have intramolecular H-bonds and exhibit a larger spectral enhancement as compared to conformers 1c and 1d.

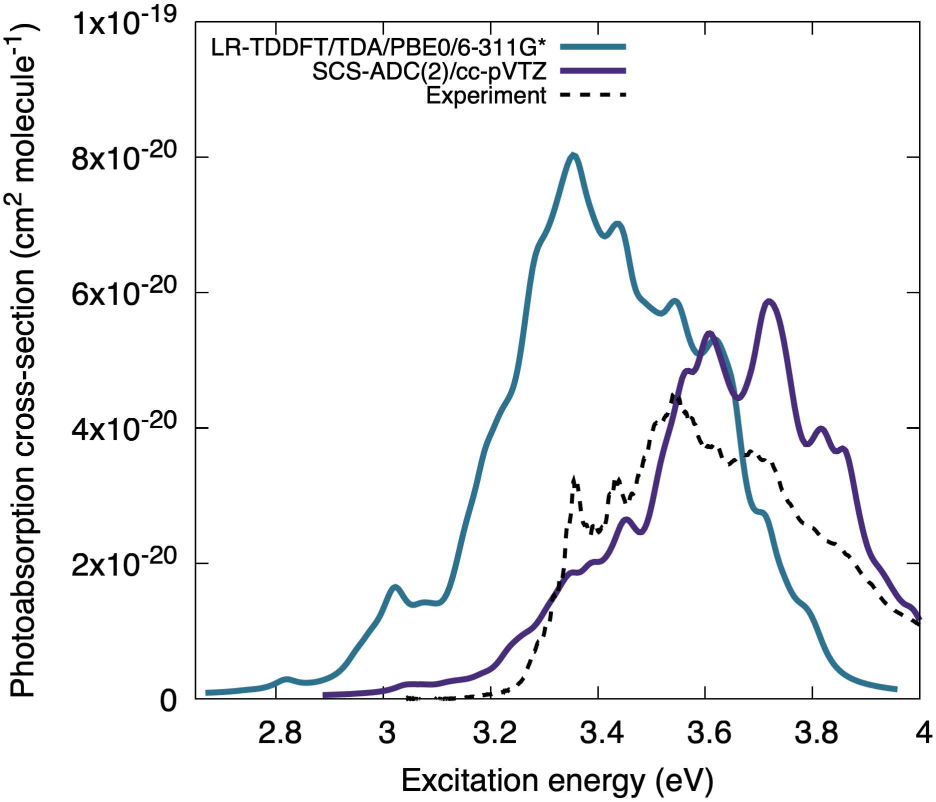

Figure S5: Experimental and calculated photoabsorption cross-sections of pyruvic acid using the NEA with a Wigner sampling. Data obtained with LR-TDDFT are compared to data from spin-component scaled algebraic diagrammatic construction of second order (SCS-ADC(2)[2]) with a cc-pVTZ basis set. SCS-ADC(2)/cc-pVTZ excited states were calculated with Turbomole 7.3.1.[3]

References

(1) Keller-Rudek, H.; Moortgat, G. K.; Sander, R.; Sörensen, R. The MPI-Mainz UV/VIS spectral atlas of gaseous molecules of atmospheric interest. *Earth Syst. Sci. Data* **2013**, *5*, 365–373.

(2) Dreuw, A.; Wormit, M. The algebraic diagrammatic construction scheme for the polarization propagator for the calculation of excited states. *Wiley Interdiscip. Rev. Comput. Mol. Sci.* **2015**, *5*, 82–95.

(3) Furche, F.; Ahlrichs, R.; Hättig, C.; Klopper, W.; Sierka, M.; Weigend, F. Turbomole. *Wiley Interdiscip. Rev. Comput. Mol. Sci.* **2014**, *4*, 91–100.
